# Development of an item pool for a questionnaire on the psychosocial consequences of hypertension labelling

**DOI:** 10.1186/s41687-019-0168-4

**Published:** 2019-12-31

**Authors:** János Valery Gyuricza, Ana Flávia Pires Lucas d’Oliveira, Lucas Bastos Marcondes Machado, John Brodersen

**Affiliations:** 1 Departamento de Medicina Preventiva, Faculdade de Medicina da Universidade de São Paulo, Av. Dr Arnaldo, 455 2o andar. CEP, São Paulo, SP 01246-903 Brazil; 2 Hospital das Clínicas da Faculdade de Medicina da Universidade de São Paulo, Rua Dr. Ovídio Pires de Campos, 225. CEP, São Paulo, SP 05403-010 Brazil; 3 Forskningsenheden for Almen Praksis, Center for Sundhed og Samfund, Københavns Universitet, Øster Farimagsgade, Bygning 24. Postboks 2099, 1014 København K, Denmark

**Keywords:** Psychosocial consequences, Patient-reported outcome measure, Hypertension

## Abstract

**Background:**

Hypertension is the most prevalent risk factor for cardiovascular disease globally. Roughly one-third of the adult population has hypertension. However, most people diagnosed with hypertension do not benefit from blood pressure control with pharmacologic interventions: they are overdiagnosed and overtreated and might experience negative psychosocial consequences of being labelled. These consequences are relevant outcomes that need to be assessed and validly measured to identify all benefits and harms related to interventions designed to prevent cardiovascular disease.

**Objectives:**

To develop a pool of items with high content validity for a draft version of a condition-specific questionnaire to measure the psychosocial consequences of being labelled with mild hypertension.

**Methods:**

We selected relevant items from existing Consequences of Screening (COS) questionnaires. These items belonged to two groups: COS core items and potential condition-specific items. All items were originally in Danish and were translated into Brazilian Portuguese using the dual-panel method. Individual and group interviews were conducted with people with mild hypertension and low risk for cardiovascular disease, and were designed to test the translated items for face and content validity and were also used to generate new relevant items. Structured individual interviews were conducted to categorise all the items into domains.

**Results:**

The Brazilian Portuguese dual-panel translation of both groups of items was found to be relevant for adults diagnosed with hypertension. We generated 52 new items to achieve high content validity. The result was a set of 132 items divided into 22 domains in 2 parts. Part I was directed at the general population, whereas part II was directed only at people diagnosed with hypertension and it consisted of 38 items in 8 domains. Twelve items remained as single items. High content validity was achieved with the pool of 132 items divided into 22 domains in 2 parts.

**Discussion:**

High content validity was achieved for a condition-specific questionnaire measuring the psychosocial consequences of being labelled with mild hypertension. This instrument encompassed 132 items divided into 22 domains in 2 parts. Thereby, a draft of the Consequneces of Hypertension questionnaire (COH) was developed. The psychometric properties of this questionnaire will be discussed in a diferent paper.

## Background

Hypertension is the most prevalent risk factor related to the development of cardiovascular disease. It is in most cases an asymptomatic condition, and its diagnosis is dependent on preventive strategies directed at the general population or at patients visiting the doctor. Currently, hypertension is defined in Brazil as blood pressure averages above 140/90 mmHg [1], divided in three stages, as shown in Table 1.

**Table 1:** Hypertesion Classification in Brazil

Classification	Systolic (mmHg)	Diastolic (mmHg)
Normal	< 120	< 80
Pre-hypertension	121–139	81–89
Stage 1 hypertension (mild)	140–159	90–99
Stage 2 hypertension (moderate)	160–179	100–109
Stage 3 hypertension (severe)	> 179	> 109

When implementing preventive health strategies, both intended benefits and unintended harms are expected. These two sides can be counterweighed in various ways, but benefits and harms are multidimensional [2] and are not always easily understood and quantified [3]. In the case of hypertension, the benefits in terms of morbidity and mortality are clear to those patients in the moderate and high-risk groups (moderate and severe hypertension); however, these patients represent only about one-third of all people diagnosed with hypertension, while the other two-thirds have mild hypertension.

Screening for hypertension is controversial. One good-quality randomized controlled trial of 140.642 people of blood pressure screening program, [4] suggested that there were 3 fewer annual hospital admissions for cardiovascular disease per 1000 persons in the intervention group. On the other hand, also in the intervention group, new antihypertensive prescription was 10% higher.

From a clinical epidemiology perspective, the thresholds defined in guidelines for hypertension have also been controvertial [5–7]. Due to the nature of the relationship between blood pressure and cardiovascular disease, there is an uncertainty range in which the threshold could be set [8]. Gudelines have evolved from defining hypertension with a few measures to including more specific diagnostic tools, such as home blood pressure monitoring for longer periods of time [9]. However, guidelines have tended to lower hypertension definitions [10], besides the lack of evidence to do so [11].

Best available evidence shows that pharmacological treatment for mild hypertension has not been established [12, 13]. A Cochrane review on pharmacotherapy for mild hypertension concludes that antihypertensive drugs used in the treatment of adults with mild hypertension have not been shown to reduce mortality or morbidity in randomized controlled trials, while 9% of patients discontinued treatment due to adverse effects. More recently, a longitudinal cohort found no evidence to support the initiation of treatment in patients with mild hypertension. This cohort lasted from 1998 til 2015 and included 19.143 adults who had mild hypertension without comorbidities (low-risk mild hypertension) and no previous treatment.

In addition, the side effects of anti-hypertensive treatment for cardiovascular disease have no relationship with the severity of the baseline condition and are equally distributed on the continuum of hypertension. This equal distribution means that those individuals with mild hypertension have the same risk of unintentional harm when compared with those with moderate to severe hypertension [14].

Moreover, evidence suggests that during the diagnosis process, people are harmed in various other ways besides the typical side effects of medication. This harm can be conceptualised as the psychosocial consequences of being labelled with a diagnosis.

Few qualitative studies have been conducted among people with hypertension to assess aspects related to psychosocial consequences [15, 16]. One study describes the interviews with 27 patients with hypertension who referred to hospitals. They experienced many physical, psychological, social, familial and spiritual problems which were associated with hypertension. Another study interviewed 6 hospitalized patients undergoing clinical investigation related to hypertension, who described the impact of the diagnosis in their daily lives. However, these two studies did not address people with mild hypertension without comorbidities in a community setting for the psychosocial consequences of labelling hypertension.

One Danish study included people with mild hypertension in a primary healthcare setting and concluded that: the diagnosis of hypertension is a biographical disruption and impacts on daily life and patients’ adaptation to hypertension combines biographical and bodily experiences [17].

Recently, we conducted a qualitative study assessing the psychosocial consequences of labelling people in Brazil with mild hypertension. We found that the diagnosis of mild hypertension is a significant event that affects daily life, and most of the impact is regarded by patients as having negative psychosocial consequences or causing harm [18].

These psychosocial consequences have also been the subject of quantitative interventional studies from a medical perspective. The bulk of evidence points to poorer interpersonal relationships, greater absenteeism and increased healthcare service use, among others, as consequences of being labelled as hypertensive [19–25].

Other studies have used instruments designed to assess health-related quality of life and psychological distress, mainly using the SF-12 and SF-36 with people with hypertension [26]. However, these generic instruments potentially lack content validity: they address topics that are irrelevant to individuals with hypertension and do not comprehensively address all relevant topics [27–29].

Furthermore, psychosocial aspects of life are typically assessed via patient-oriented perspectives. Specific questionnaires have been developed and used to assess quality of life in people with hypertension (e.g. CHAL and MINICHAL). However, these questionnaires were not developed from the patients’ perspective; thus, they potentially also lack content validity [30–33].

More recently, with patient-centred medicine helping to balancing the doctor and the patient perspectives, Patient Reported Outcome Measures (PROMs) emerged [34] with items used to measure psychosocial attributes oriented by the patients’ perspectives [35]. These perspectives can be assessed qualitatively to generate items, which are later tested for content validity to create a draft questionnaire that can then be investigated for its psychometric properties [36].

An example of this type of questionnaire is the Consequences of Screening (COS) questionnaire [37–40]. COS is a family of questionnaires addressing various screening scenarios for life-threatening diseases, which is not the case of hypertension. However, it has been shown in qualitative studies that in spite of these differences in severity, living with life-threatening diseases share similarities to living with hypertension [41]. The first questionnaire of this series was developed to capture the psychosocial consequences of abnormal and false-positive screening mammography for breast cancer and was named the COS-BC [37]. Later, additional versions were developed to address other screening scenarios for life-threatening, non-communicable diseases, including lung cancer, abdominal aortic aneurism and cervical cancer [38–40]. The COS questionnaires were developed in Danish and have a two-part common core questionnaire, in which the first part measures the negative psychosocial consequences at any time during the screening process, while the second part assesses changes in the long-term psychosocial consequences of screening after a final diagnosis.

We hypothesised that if people diagnosed with mild hypertension regarded this diagnosis as a life-threatening disease, then at least the core items and dimensions and possibly some of the condition-specific COS items would be relevant to people labelled with mild hypertension. Therefore, the overall purpose of this study was to develop a pool of items for a condition-specific draft questionnaire with high content validity to measure the psychosocial consequences of being diagnosed with mild hypertension. The steps to reach this aim were as follows:

1. Conduct a systematic literature search for questionnaires other than COS that address issues in hypertension and patient-oriented outcomes in terms of the consequences of labelling;
2. Translate and adapt all the COS core items and all the relevant condition-specific items from other COS questionnaires into Brazilian Portuguese;
3. Assess the content relevance and content coverage of the Brazilian Portuguese core and condition-specific items in patients diagnosed with mild hypertension;
4. Generate new condition-specific domains and items especially relevant for patient diagnosed with mild hypertension if a lack of content coverage of the Brazilian Portuguese core and condition-specific COS items was revealed;
5. Assess all instructions and items for functionality and understandability.

Later, this pool of items will be tested in a survey, and have their psychometric properties assessed. The psychometric results will be published later.

## Methods

To assess face validity, a systematic literature search was conducted in Medline and PsycINFO for articles in English and Portuguese to identify questionnaires used to assess the psychosocial consequences of being diagnosed with hypertension. We used a broad set of search terms related to hypertension (high blood pressure, blood pressure, arterial pressure, hypertension and risk factor); labelling (diagnosis, stereotyping, stigma and awareness) and PROMs (quality of life, patient outcomes, surveys, questionnaires and patient-reported outcome measures). We selected the questionnaires that suited our needs and then cherry picked all items that seemed relevant.

### Dual panel

We translated the all relevant items from the selected questionnaires into Brazilian Portuguese using the dual-panel method [42].

#### Bilingual panel

First, in São Paulo, we conducted a bilingual panel including two researchers and four people who were bilingual (fluent in Danish and with mother tongue in Brazilian Portuguese). The panel members were asked to translate all instructions and items from Danish into Brazilian Portuguese. If there were divergences in the translations, they were asked to discuss and find a consensual translation. If the panel members could not reach consensus they were allowed to generate two or more versions and leave it up to the next panel to decide which translation was most close to lay Brazilian Portuguese language.

#### Lay panel

Second, the lay panel included people living in São Paulo and who had no knowledge of the Danish language. The members of the lay panel were five community healthcare workers (CHW). In addition, one of the bilingual experts helped JB during the lay panel. He translated discussions and questions from Portuguese into Danish and vice-versa. The translated items were read together with the group, and we asked if the versions produced by the bilingual panel were expressed in easily understandable lay language. After this session, a draft of the Consequences of Hypertension (COH) questionnaire in Brazilian Portuguese was drafted.

### Interviews

The recruitment of informants has previously been described in details [18]. In short, we recruited the informants for interviews ad hoc from public primary healthcare services in São Paulo (known as Unidade Básica de Saúde-UBS), social media and social networks. They were strategically recruited to obtain a wide range of experiences and ages, times from diagnosis, education levels and ethnic groups and both sexes. People recruited from the UBS were identified from the list of people diagnosed with hypertension. People recruited via social media responded to an invitation posted on Facebook. Two of the informants were recruited via the researchers’ own social network. A telephone interview was conducted prior to the face-to-face interviews to assess inclusion and exclusion criteria.

Inclusion criteria: Raised in Brazil, confirmed diagnosis of mild hypertension by a physician, prescribed anti-hypertensive treatment and with no other chronic or disabling conditions.

All interviews were digitally audio recorded and transcribed verbatim.

#### In-depth individual interviews

Eleven 1–2 h semi-structured, individual in-depth interviews were conducted between October 2016 and March 2017 at a location the informants found least inconvenient (Table 4 in Appendix).

After the in-depth interviews, the informants completed the draft COH during a think-aloud session [43]. They were asked to formulate opinions on the instructions, on the items and on the layout of the questionnaire.

Later, we read and discussed the content of the interviews; if lack of content coverage was identified, we formulated new items that reflected informants’ verbatim expressions (whenever possible), categorised the items into previous domains and suggested new domains when new items did not fit into the previous domains. These new domains and items were then added to the COH for the next steps.

#### Focus groups

Next, we conducted four 2-h focus-group interviews in an easy-to-access location (Table 4 in Appendix). The informants included in these interviews were grouped strategically with similar characteristics regarding sex and education level.

The focus-group interviews consisted of two parts: first, we led an open-ended discussion for about 30 min, and for the next 90 min, we discussed the draft COH questionnaire. All the items were tested, but we first focused on the newly generated items. We asked the group if the items were understandable, represented experiences that they might have had (content relevance) and if there were any domains or items missing or irrelevant (content coverage).

#### Structured interviews

Finally, we conducted four 60-min structured individual interviews. (Table 4 in Appendix) The informants were given a list of all items and asked to elaborate on all of the new condition-specific items and to categorise them into pre-determined domains.

Given that recently elaborated items were tested, the informants were told they could be categorised into one of the existing domains, or if necessary, a new domain could be suggested. Similar suggestions on an item were considered powerful enough to categorize that item or to lead to the creation of a new domain, while items without similar suggestions were left for later discussion among the authors.

## Results

### Literature search

No condition-specific PROM on the consequences of labelling people with hypertension was identified. Therefore, the COS questionnaires were chosen as the only relevant source of items.

We selected 76 items (55 items from part I and 21 from part II) from the 4 COS questionnaires; Half of which (26 from part I and 12 from part II) are present in all COS questionnaires and compose the core items. The other half (29 from part I and 9 from part II) is present in COS as disease specific items.

**Fig. 1:**
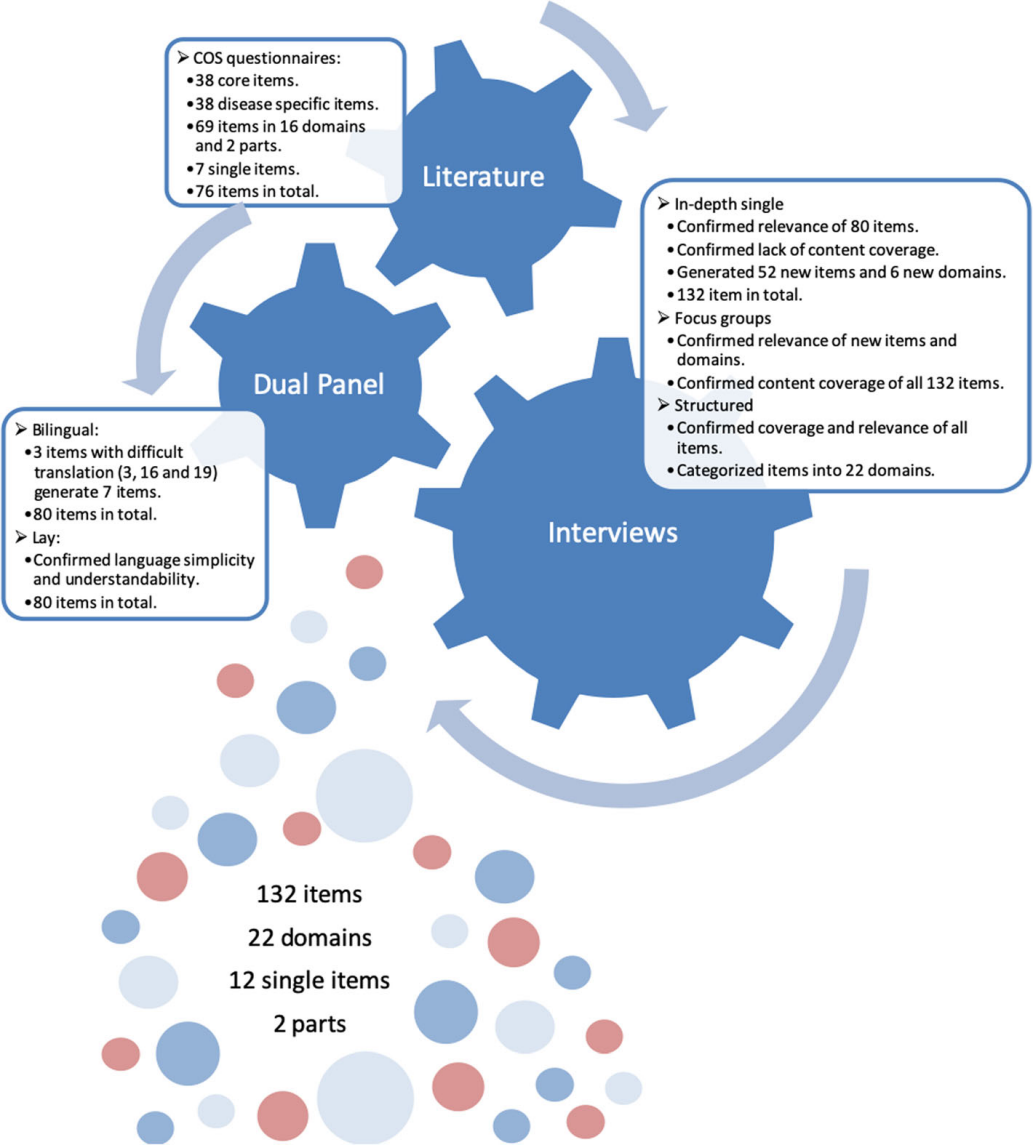
Material, Methods and Results

A total of 69 items out of these 76 items were representative of 17 different domains: 12 in part I and 5 in part II, while 7 items were regarded as single items: 2 in part I and 5 in part II. Table 2 lists all items, with their respective Brazilian Portuguese wordings, domains, parts, positions, origins, meaning in English or Danish and response categories. Figure 1 describes all the methodological steps and results of the present study.

**Table 2:** All items in Brazilian Portuguese and the ad hoc translation. Items in part II are sentences completed with the response categories provided. For example: item 97, which is ‘my joy of living became…’ can be completed with ‘the same as before’. Item 95 (‘do you have high blood pressure? yes/no’) is not included in the list. This item will be used to determine those who are required to complete part II

Part	Position	Brazilian Portuguese version	Questionnaire of origin	Domain	ad hoc English translation
I	1	Me senti preocupado	core	Sense of dejection	I felt worried
I	2	Me senti preocupado com meu futuro	core	Anxiety	I felt worried about my future
I	3	Me senti amedrontado	core	Anxiety	I felt frightened
I	4	Me senti com medo	core	Anxiety	I felt scared
I	5	Me senti irritado	core	Behaviour	I felt annoyed
I	6	Me senti mais quieto do que o normal	core	Behaviour	I felt quieter than usual
I	7	Dormi mal à noite	core	Sleep	I slept badly at night
I	8	Fuji dos meus pensamentos me ocupando com tarefas práticas do dia-a-dia	core	Single Items	I ran away from my thoughts, busy with day-to-day practical tasks
I	9	Me senti com dificuldade de me concentrar	core	Behaviour	I felt hard to concentrate
I	10	Me senti com a sensação de que o tempo não passava	core	Sense of dejection	I felt that time was not passing
I	11	Tive mudanças em meu apetite	core	Behaviour	I had changes in my appetite
I	12	Me senti triste	core	Sense of dejection	I felt sad
I	13	Me senti emotionalmente fora do meu normal	core	Anxiety	I felt emotionally out of my normal
I	14	Me senti inquieto	core	Anxiety	I felt restless
I	15	Me senti nervoso	core	Anxiety	I felt nervous
I	16	Me senti ansioso	core	Anxiety	I felt anxious
I	17	Tive dificuldade de pegar no sono	core	Sleep	I had difficulty falling asleep
I	18	Me senti mais fechado	core	Behaviour	I felt introvert
I	19	Me senti sem iniciativa	core	Sense of dejection	I felt without initiative
I	20	Me senti sem vontade	core	Sense of dejection	I felt unwilling
I	21	Me senti deprimido	core	Sense of dejection	I felt depressed
I	22	Tive dificuldades em realizar meu trabalho e outras tarefas semelhantes	core	Behaviour	I had difficulty doing my job and other similar tasks
I	23	Acordei cedo demais	core	Sleep	I woke up too early
I	24	Tive dificuldades em realizar tarefas de casa	core	Behaviour	I had difficulty doing domestic work
I	25	Me senti a ponto de entrar em pânico	core	Anxiety	I felt about to panic
I	26	Passei a maior parte do tempo acordado	core	Sleep	I spent most of the time awake
I	27	Tive menos desejo sexual	core	Sexual	I had less sexual desire
I	28	Dias faltados no trabalho	core	Single Items	Days missed at work
I	29	Me senti em estado de choque	disease specific	Anxiety	I felt in shock
I	30	Fiquei com medo da pressão alta o tempo todo na cabeça	new	Blood pressure related	I had the fear of high blood pressure all of the time in the head
I	31	Me senti inseguro	disease specific	Introvert	I felt insecure
I	32	Me senti com pena de mim mesmo	disease specific	Introvert	I felt sorry for myself
I	33	Me senti em uma situação desesperadora	disease specific	Introvert	I felt in a desperate situation
I	34	Fiquei com humor muito variável	disease specific	Introvert	I was in a very variable mood
I	35	Me senti mais cansado do que de costume	disease specific	Single Items	I felt more tired than usual
I	36	Guardei meus pensamentos só pra mim	disease specific	Single Items	I kept my thoughts just for myself
I	37	Me senti doente	disease specific	Body Perception	I felt sick
I	38	Tive a sensação de que havia algo errado com meu corpo	disease specific	Body Perception	I had a feeling something was wrong with my body
I	39	Me senti fora de controle	disease specific	Fear and Powerlessness	I felt out of control
I	40	Me senti com o corpo frágil	disease specific	Fear and Powerlessness	I felt my body fragile
I	41	Senti que a idade chegou	disease specific	Perception of age	I felt that old age has come
I	42	Me senti como se meu corpo fosse uma máquina que não funciona	disease specific	Body Perception	I felt like my body was a non-working machine
I	43	Me senti azedo	disease specific	Emotional	I felt sour
I	44	Me senti zangado	disease specific	Emotional	I felt angry
I	45	Me senti como se estivesse no vazio	disease specific	Single Items	I felt like I was in the void
I	46	Me senti como um estranho em meu próprio corpo	disease specific	Body Perception	I felt like a stranger in my own body
I	47	Me senti mais velho do que sou	disease specific	Perception of age	I felt older than I am
I	48	Me senti sem forças	disease specific	Fear and Powerlessness	I felt strengthless
I	49	Chorei mais do que de costume	disease specific	Emotional	I cried more than usual
I	50	Me senti sem sorte	disease specific	Fear and Powerlessness	I felt unlucky
I	51	Me senti vulnerável	disease specific	Fear and Powerlessness	I felt vulnerable
I	52	Me senti fragilizado	disease specific	Single Items	I felt weak
I	53	Me senti como se qualquer coisa pudesse me afetar	disease specific	Body Perception	I felt like anything could affect me
I	54	Mudei meus hábitos de atividade física	disease specific	Lifestyle	I changed my exercising habits
I	55	Pensei na morte	disease specific	Single Items	I thought about death
I	56	Mudei meus hábitos alimentares	disease specific	Lifestyle	I changed my eating habits
I	57	Pensei que seria melhor se não soubesse que tenho pressão alta	new	Blood pressure related	I thought it would be better if I didn’t know I have high blood pressure
I	58	Tive medo de fazer esforço físico	disease specific	Fear and Powerlessness	I was afraid of doing exercises
I	59	Me senti insatisfeito com minha vida sexual	disease specific	Sexual	I felt dissatisfied with my sex life
I	60	Pensei na minha fé	new	Single Items	I thought of my faith
I	61	Me senti impaciente	new	Anxiety	I felt impatient
I	62	Me senti culpado	new	Sense of dejection	I felt guilty
I	63	Me senti desequilibrado	new	Emotional	I felt unbalanced
I	64	Senti que não tenho saúde	new	Body Perception	I felt that I am not healthy
I	65	Me senti em dúvida	new	Results of diagnosis	I felt in doubt
I	66	Me senti sem saber o que esperar	new	Fear and Powerlessness	I didn’t know what to expect
I	67	Me senti desmotivado	new	Sense of dejection	I felt unmotivated
I	68	Me senti desestimulado	new	Sense of dejection	I felt discouraged
I	69	Me senti fraco	new	Body Perception	I felt weak
I	70	Me senti frustrado	new	Sense of dejection	I felt frustrated
I	71	Me senti indiferente	new	Sense of dejection	I felt indifferent
I	72	Me senti sendo julgado	new	Social Relations	I felt being judged
I	73	Me senti com pavor	new	Fear and Powerlessness	I felt terrified
I	74	Me senti preso	new	Emotional	I felt trapped
I	75	Me senti sendo forçado a fazer coisas que não quero	new	Single Items	I felt being forced to do things I don’t want
I	76	Me senti orgulhoso	new	Emotional	I felt proud
I	77	Me senti apreensivo	new	Fear and Powerlessness	I felt apprehensive
I	78	Me senti com raiva	new	Emotional	I felt angry
I	79	Me senti impotente	new	Fear and Powerlessness	I felt helpless
I	80	Me senti surpreso	new	Results of diagnosis	I felt surprised
I	81	Me senti tranquilo	new	Single Items	I felt calm
I	82	Me senti chateado	new	Sense of dejection	I felt upset
I	83	Me senti envergonhado	new	Emotional	I felt ashamed
I	84	Me senti controlado pelos outros	new	Social Relations	I felt controlled by others
I	85	Me senti apoiado	new	Social Relations	I felt supported
I	86	Me senti excluído	new	Social Relations	I felt excluded
I	87	Me senti cuidado	new	Social Relations	I felt being cared for
I	88	Me senti diferente	new	Social Relations	I felt different
I	89	Me senti importante	new	Social Relations	I felt important
I	90	Tive sintomas de pressão alta	new	Blood pressure related	I had symptoms of high blood pressure
I	91	Me senti culpado por não cuidar de mim mesmo como deveria	new	Sense of dejection	I felt guilty for not taking care of myself as I should
I	92	Me senti assustado	new	Fear and Powerlessness	I felt scared
I	93	Me senti agitado	core	Anxiety	I felt agitated
I	94	Me senti incomodado	core	Sense of dejection	I felt bothered
II	96	eu fiquei pensando na vida	core	Existential values	I kept thinking about life
II	97	minha alegria de viver ficou	core	Existential values	my joy of living became…
II	98	me senti tranquilo	core	Relaxed/Calm	I felt calm
II	99	a minha relação com a minha família ficou	core	Personal Relations	my relationship with my family became…
II	100	a minha relação com meus amigos ficou	core	Personal Relations	my relationship with my friends became…
II	101	a minha relação com outras pessoas ficou	core	Personal Relations	my relationship with other people became…
II	102	me senti calmo	core	Relaxed/Calm	I felt calm
II	103	a minha visão do futuro ficou	core	Existential values	my vision of the future became…
II	104	a minha sensação de bem-estar ficou	core	Existential values	my sense of well-being became…
II	105	a minha percepção sobre a vida ficou	core	Existential values	my perception of life became…
II	106	o valor que dou a vida ficou	core	Existential values	the value I give in life became…
II	107	a minha energia ficou	disease specific	Impulsive	my energy became…
II	108	meu sentimento de responsabilidade pela minha família ficou	disease specific	Empathy	my sense of responsibility for my family became…
II	109	tenho aproveitado a vida	disease specific	Impulsive	I have enjoyed life
II	110	me sinto aliviado	core	Relaxed/Calm	I feel relieved
II	111	minha compreensão dos problemas alheios ficou	disease specific	Empathy	my understanding of other people’s problems became…
II	112	me sinto impulsivo	disease specific	Impulsive	I feel impulsive
II	113	a minha capacidade de ouvir problemas alheios ficou	disease specific	Empathy	my ability to hear other people’s problems became…
II	114	a minha vontade de me envolver com algo novo ficou	disease specific	Impulsive	my desire to get involved with something new became…
II	115	a minha vontade de me envolver com algo arriscado ficou	disease specific	Impulsive	my desire to get involved with something risky got…
II	116	tenho feito coisas que utrapassam meus limites	disease specific	Impulsive	I’ve been doing things that push my limits
II	117	frequento consultas médicas	new	Patient Role	I go to doctor’s appointments
II	118	faço exames	new	Patient Role	I take exams
II	119	me sinto fazendo mal para mim mesmo	new	Patient Role	I feel bad for myself
II	120	me sinto com dificuldades em seguir orientações médicas	new	Patient Role	I have difficulty following medical advice
II	121	me sinto cuidando de mim mesmo	new	Patient Role	I feel taking care of myself
II	122	tomo medicamentos	new	Patient Role	I take medicines
II	123	me sinto dependente de remédios	new	Patient Role	I feel dependent on medicines
II	124	me sinto confiante em orientações médicas	new	Patient Role	I feel confident in medical advice
II	125	me sinto como se não fosse mais normal	new	Existential values	I feel like I’m not normal anymore
II	126	me sinto como se não fosse mais o mesmo	new	Existential values	I feel like I’m not the same anymore
II	127	me sinto preocupado com sintomas de pressão alta	new	Preoccupation with health	I feel worried about symptoms of high blood pressure
II	128	me sinto preocupado com meus hábitos e estilo de vida	new	Preoccupation with health	I feel worried about my habits and lifestyle
II	129	me sinto preocupado com os tratamentos	new	Preoccupation with health	I feel worried about the treatments
II	130	meu desempenho no trabalho ficou	new	Single Items	my work performance became…
II	131	minha prática sexual ficou	new	Single Items	my sexual practice became…
II	132	minha ansiedade com relação a pressão alta ficou	new	Hypertension related	my anxiety about high blood pressure got
II	133	penso que eu não tenho pressão alta	new	Hypertension related	I think I don’t have high blood pressure

### Dual panel

Three 76 core items generated more than 1 version in Brazilian Portuguese, resulting in a total of 80 items.

#### Bilingual panel

All original Danish items, except three from part I, did reach consensual Brazilian translation. These three items were given more than one Brazilian version: Items 16, 93 and 94 were three Brazilian items representing different translated and adaptated versions of the original Danish item 16 (‘I felt bothered’); items 19 and 20 are two versions of original Danish item 19 (‘I felt paralyzed’); and items 3 and 4 are also two Brazilian versions of the original Danish item 3 (‘I felt scared’). Therefore, after conducting this panel, the 76 original Danish items became 80 Brazilian items.

#### Lay panel

The group confirmed the instructions’ and items’ translations as lay language and understandable. They were unable to select one item out of the versions for items 3, 16 and 19, and therefore all were kept, confirming all 80 items. One sensitive suggestion was confirmed during this part, which was related to the inversion of the pronominal preposition in Brazilian Portuguese. Although this represents a grammatically incorrect form of the sentences, it is directly related to the way Brazilian people speak. All the sentences were then rewritten from ‘Senti*-me…’* to ‘*Me senti …’*.

### Interviews

Altogether we included in all three kinds of interviews 27 informants of both sexes, aged 21–74 years, being diagnosed with hypertension 1 month to 30 years ago, education level low to high, including illiteracy, and various ethnic groups.

Our informants content-validated the 80 translated items. In total, we generated 52 new items (35 for part I and 17 for part II) for 12 domains. Twenty-five of these new were encompassed in 6 new domains. In part I 3 new domains were generated: the ‘blood pressure-related’ domain encompassing 3 items, the ‘social relations’ domain encompassing 7 items and the ‘results of diagnosis’ domain encompassing 2 items. In part II 3 new domains were also generated: the ‘hypertension-related’ domain encompassing 2 items, the ‘patient role’ domain encompassing 8 items and the ‘preoccupation with health’ domain encompassing 3 items.

#### In-depth individual interviews

##### Instructions

Three options of instructions for part I were designed based on our previous experiences with questionnaires. We offered our informants these three options and asked them to elaborate on them:

- How have you been feeling the last month? (*Como você se sentiu no ultimo mês?*) (Or)
- How have you been feeling the last week? (*Como você se sentiu no ultimo semana?*) (Or)
- How do you feel nowadays regarding blood pressure? (*Como você se sente hoje em dia com relação à pressão?)*

The informants suggested that the best way to frame the instruction of part I was the first option: ‘how have you been feeling the last month?’, and we chose this one for the questionnaire. They suggested that the second option included a too short of a period, while the third was rejected beacause it was too broad.

Complementary part II was opened with the question: Taking everything into account: the diagnosis, the follow-up, the exams, the pills… (*Levando tudo em consideração: o diagnostico, o seguimento, os exames, os remédios …*); and part II has items introduced by the sentence: … after I knew I had high blood pressure … (… *depois de saber que tenho pressão alta* …). No changes were suggested in this part.

##### Response categories

The original COS was developed with polytomous items. Part I had the following possible answers:

- No, not at all/no, not even once (*não, nem um pouco/não, nem uma vez*)
- Yes, a little/yes, a few times (*sim, um pouco/sim, poucas vezes*)
- Yes, some/yes, sometimes (*sim, não muito/sim, às vezes*)
- Yes, a lot/yes, many times (*sim, muito/sim, muitas vezes*)

A few items had a fifth option: I don’t know (*não sei*), and one item was relevant to counting the number of missing days at work and had the option: 0, 1–2, 3–4 or 5 or more; I don’t work. These response categories were confirmed to be relevant, comprehensive, understandable and easy to complete.

The same was found for the translation of the original response categories in part 2. All items were polytomous, with the following possible answers:

- A lot less… *(muito menos)*
- Some less… *(um pouco menos)*
- The same as before…*(o mesmo que antes)*
- Some more… *(um pouco mais)*
- A lot more…*(muito mais)*

#### Focus groups

No new items were developed. The groups confirmed high content validity of the 132 items.

#### Structured interviews

Five new items (3 from part I and 2 from part II) could not be categorised by the informants into any of the existing domains and were therefore regarded as single items (Table 3). We also asked the informants to allocate the versions of the two original items without a consensual translation to a domain: items 16, 93 and 94 (originally item 16) and items 19 and 20 (originally item 19). Items 16 and 93 were categorised in a different domain (‘anxiety’) compared to item 94 that stayed in the original domain (‘sense of dejection’). Items 19 and 20 were both categorised as belonging to the domain of ‘sense of dejection’.

**Table 3:** Dimensions and items

	Number of items
Part I	94
Anxiety	11
core	9
disease specific	1
new	1
Behaviour	7
core	7
Blood pressure related	3
new	3
Body Perception	7
disease specific	5
new	2
Emotional	8
disease specific	3
new	5
Fear and Powerlessness	11
disease specific	6
new	5
Introvert	4
disease specific	4
Lifestyle	2
disease specific	2
Perception of age	2
disease specific	2
Results of diagnosis	2
new	2
Sense of dejection	14
core	7
new	7
Sexual	2
core	1
disease specific	1
Single Items	10
core	2
disease specific	5
new	3
Sleep	4
core	4
Social Relations	7
new	7
Part II	38
Empathy	3
disease specific	3
Existential values	8
core	6
new	2
Hypertension related	2
new	2
Impulsive	6
disease specific	6
Patient Role	8
new	8
Personal Relations	3
core	3
Preoccupation with health	3
new	3
Relaxed/Calm	3
core	3
Single Items	2
new	2
Total Geral	132

## Discussion

### Major findings

To achieve high content validity of a measure about psychosocial consequences of being diagnosed with mild hypertension we included a total of 132 items divided into 22 domains in 2 questionnaire parts: Part I encompassed 94 items in 14 domains, part II 38 items in 8 domains.

Ten items remained as single items in part I and two remained in part II. Although a single item does not necessarily have a high measurement precision like a scale, it could be wise to keep these items for content coverage because if a single item has high relevance informants might interprete a questionnaire without such single items as having lack of content coverage: they think important questions are missing.

We did not find any previously published PROMs addressing the psychosocial consequences of labelling people with mild hypertension in our literature search. Qualitative studies describe similar experiences in people living with cancer and people living with cardiovascular disease [40]. Moreover, one of the authors has previously developed the COS questionnaires. The use of previously developed items could be a fast way to the development of new scales, saves time and money and is a common practice: the COS itself was based on previously developed items [37]. We selected the COS questionnaires for the following reasons: accessibility to the content, plausible similar psychosocial consequences between false positives and overdiagnosed in a screening context, the diagnosis of a chronic condition and already established psychometric properties of COS (in Danish and Swedish).

Our choice of translation method was based on its prior use in the development of many other disease-specific measures in up to 30 languages [44]. Recruiting CHWs as informants for the lay panel was found to be a strength since they have a broad social network and a wide range of cultural experiences and are similar to the target of this questionnaire.

We have generated a very large item pool. This seems like a weakness of this study, since a very long questionnaire might have limited use. However, this is one of the strengths of this study, because it provides a broad range of items for every domain. This broad range of items describes different nuances and will provide enough elements for the psychometric analysis of each domain. It is expected that after the psychometric analysis, the item pool will be significantly reduced.

Face validity was confirmed in the interviews; however, numerous new items had to be added to achieve high content validity of the COH. Another strength of our study is the population for the interviews, which included informants with a broad range of sociodemographic characteristics including health professionals. All of them were residents of São Paulo, which might be a limitation. However, many were migrants from other Brazilian regions. Moreover, we conducted a qualitative study on the psychosocial consequences of being labelled with mild hypertension, and achieved data saturation before conducting any of the group interviews, which might indicate that we had achieved high content coverage for most of the psychosocial consequences of being labelled with mild hypertension.

We asked our informants in single interviews to evaluate 80 translated items from the COS. All the items were found to be relevant and were included in the final draft of the questionnaire. This result might indicate that patients living with the diagnosis of mild hypertension share similarities with those experiencing abnormal results in screening for cancer and abdominal aortic aneurism – diseases that are regarded by most lay people as deadly life-threatning diseases with poor prognoses.

The fact that 52 new items and 6 new domains emerged from our qualitative study indicates that the COS were not comprehensive in a context of mild hypertension. Most of the items were derived directly from transcriptions of words or sentences from the informations verbatim expressions. However, a few were generated based on our analyses of the meaning condensation of the interviews [18]. One example is the item on pride. No informant used the word pride to refer to their experiences, but we noted a sense of pride in their statements referring to efforts and achievements in controlling hypertension and complying with medical prescriptions. The wording of this pride item and other items were confirmed in the following focus-group interviews.

The methods described in this article represent a consistent way to achieve high content validity for PROMs. We used three different qualitative methods because each of them had a different focus and complemented each other, which we see as a strength. Furthermore, if we attempted to address all our needs with every informant, the result would be a very tiresome interview. The purpose of the in-depth semi-structured individual interviews was to gain insight into the consequences of labelling mild hypertension, to describe the consequences of this diagnosis, and to test the COS for content validity in this setting. These interviews were also part of our qualitative study on the psychosocial consequences of labelling hypertension [18]. After that, the informants were exposed to a draft version of the questionnaire, allowing them to reflect and evaluate the instructions and the items’ content validity. A similar method was used with the focus-group interviews, where we only showed the items after the group had the opportunity for open-ended reflection to discuss and debate the psychosocial consequences of being labelled with mild hypertension.

## Conclusion

High content validity was achieved for a condition-specific questionnaire measuring the psychosocial consequences of being labelled with mild hypertension. This instrument encompassed 132 items divided into 22 domains in 2 parts. Thereby, a draft of the Consequneces of Hypertension questionnaire (COH) was developed.

## Implications for research

All domains will have to be analysed for unidimensionality and invariant measurement using primarily Item Response Theory Rasch models but also Classical Test Theory, to validate the COH and thereby select the items for the final version of the COH.

The final questionnaire is intended to be used in hypertension research, specially in hypertension screening scenarios, in which the results might bring to light the unintended psychosocial harms of labelling.

## Data Availability

The datasets used and/or analyzed during the current study are available from the corresponding author on reasonable request.

## Appendix

**Table 4:** Demographic characteristics and qualitative method fase

order	Qualitative phase	Sex	Age	Ethnic	Education (years completed)	Time since diagnosis (years)	Local
1	semi-structured individual	male	30	white	18	4	public place
2	semi-structured individual	female	21	black	9	4 months	home
3	semi-structured individual	male	36	white	19	3	work
4	semi-structured individual	female	35	mixed	8	1 month	UBS
5	semi-structured individual	female	36	white	16	4	home
6	semi-structured individual	male	46	white	11	1.5	UBS
7	semi-structured individual	female	44	white	20	9	work
8	semi-structured individual	female	64	white	11	7	home
9	semi-structured individual	female	65	white	11	22	home
10	semi-structured individual	female	64	mixed	11	15	home
11	semi-structured individual	male	44	mixed	18	8	work
1	focus group	female	42	mixed	11	12	UBS
1	focus group	female	43	white	15	2	UBS
1	focus group	female	50	black	15	15	UBS
2	focus group	female	57	mixed	8	17	UBS
2	focus group	female	65	mixed	11	30	UBS
2	focus group	female	74	white	5	20	UBS
3	focus group	male	43	black	11	3	UBS
3	focus group	male	65	mixed	8	8	UBS
3	focus group	male	65	white	illiterate	15	UBS
4	focus group	female	51	mixed	16	13	Home
4	focus group	female	55	white	11	3	Home
4	focus group	female	56	white	16	1	Home
1	structured	male	45	white	19	10	work
2	structured	female	40	white	20	4	work
3	structured	female	44	white	26	9	work
4	structured	male	40	white	20	3	public place

## Abbreviations

CHW: community healthcare workers
COH: consequences of hypertension questionnaire
COS: consequences of screening questionnaire
PROM: patient-reported outcome measure
UBS: unidade básica de saúde (primary healthcare clinic)
